# Integrated Primary Care Programs for Early Detection and Management of Child and Adolescent Mental Health Issues: A One-Year Evaluation in Two Catalonian Health Areas

**DOI:** 10.1192/j.eurpsy.2026.11164

**Published:** 2026-07-31

**Authors:** C. Diaz Tellez, R. Calvo Escalona

**Affiliations:** Hospital Clinic, Barcelona, Spain

## Abstract

**Introduction:**

Mental health disorders affect 20–40% of children and adolescents and are linked to increased risks such as suicide, injuries, and poor academic performance. Despite effective treatments, only one-third receive adequate care, and delays of 8–10 years from symptom onset to treatment are common. Early identification and intervention are crucial to prevent chronicity and reduce medicalization.

**Objectives:**

This study evaluates the effectiveness of the *Collaboration Program between Mental Health and Primary and Community Care* in two primary care areas within the Child and Adolescent Mental Health Center of Eixample (Hospital Clinic), Barcelona.

**Methods:**

A retrospective review was conducted on patients assessed through PCP between July 2024 and July 2025. Sociodemographic, clinical, and follow-up variables were analyzed using descriptive statistics, Chi-square tests, and Student’s t-tests.

**Results:**

A total of 280 patients were attended (42.9% female; mean age 10.96 ± 3.6 years). The most frequent primary diagnoses were behavioral disorders (16.5%), ADHD (14.7%), and anxiety disorders (13.2%). ADHD appeared in 32.1% of cases when considering all diagnoses, and comorbidity was present in 27.9%, significantly associated with male sex (p=0.034) and higher referral rates to Mental Health Center (p<0.001). Psychosocial complexity was identified in 29.1% of cases, correlating with depressive, trauma-related, and behavioral disorders (p<0.001).

Follow-up indicators showed that 33.6% of cases were managed within program, while 37.5% required referral to Mental Health Center, mainly for severe diagnoses such as suicidal ideation (100%), depressive disorder (75%), autism spectrum disorder (61.3%), and behavioral disorders (62.2%). Symptom remission occurred in 45.2% of cases managed in the program, with an average of 3.19 visits.

**Conclusions:**

*Collaboration Program between Mental Health and Primary and Community Care* demonstrates high effectiveness in addressing child and adolescent mental health needs at the primary care level, reducing unnecessary referrals and optimizing specialized resources. Clinical profiles associated with greater complexity—such as comorbidity, specific diagnoses, and male sex—require targeted strategies. Findings support strengthening the program and integrating child psychiatrists into primary care teams to avoid pharmacological-driven referrals.

**Disclosure of Interest:**

None Declared

